# Plant disease symptom segmentation in chlorophyll fluorescence imaging with a synthetic dataset

**DOI:** 10.3389/fpls.2022.969205

**Published:** 2022-11-10

**Authors:** Natalia Sapoukhina, Tristan Boureau, David Rousseau

**Affiliations:** ^1^ Univ Angers, Institut Agro, INRAE, IRHS, SFR QUASAV, Angers, France; ^2^ Phenotic Platform, Univ Angers, Institut Agro, INRAE, IRHS, SFR QUASAV, Angers, France; ^3^ Laboratoire Angevine de Recherche en Ingénierie des Systèmes (LARIS), Université d’Angers, Angers, France

**Keywords:** synthetic data, semantic segmentation, plant disease, precision agriculture, deep learning, computer vision

## Abstract

Despite the wide use of computer vision methods in plant health monitoring, little attention is paid to segmenting the diseased leaf area at its early stages. It can be explained by the lack of datasets of plant images with annotated disease lesions. We propose a novel methodology to generate fluorescent images of diseased plants with an automated lesion annotation. We demonstrate that a U-Net model aiming to segment disease lesions on fluorescent images of plant leaves can be efficiently trained purely by a synthetically generated dataset. The trained model showed 0.793% recall and 0.723% average precision against an empirical fluorescent test dataset. Creating and using such synthetic data can be a powerful technique to facilitate the application of deep learning methods in precision crop protection. Moreover, our method of generating synthetic fluorescent images is a way to improve the generalization ability of deep learning models.

## 1 Introduction

Being a severe environmental and health issue, the unsustainable usage of chemicals in agriculture induced to development of early disease detection methods and precision spraying (Lefebvre et al., 2015). Various non-invasive and non-destructive imaging techniques, particularly thermal, multispectral, hyperspectral, and chlorophyll fluorescence, can identify infection before visible symptoms appear (Mutka and Bart, 2015; Singh et al., 2020). Plants experiencing biotic and abiotic stress exhibit changes in chlorophyll fluorescence emission (Baker, 2008). Thus, chlorophyll fluorescence imaging (CFI) is a well-established, effective tool for comprehensively examining the development and effects of bacterial, fungal, and viral infections on leaves of many cultivated plants (Rousseau et al., 2013; Rousseau et al., 2015a; Méline et al., 2019; Pérez-Bueno et al., 2019; Méline et al., 2020; Valcke, 2021). CFI has potential use for monitoring the damaging effect of diseases on plants from the laboratory to the field scale (Ivanov and Bernards, 2016). Coupled with semantic segmentation, CFI can be deployed to target location, size, and the disease state at an early stage of its progress (Ampatzidis, 2018; Singh et al., 2020). Moreover, automatic estimation of the diseased plant area can describe the disease’s epidemiological characteristics, understand disease dynamics, assess its propagation speed, and thus facilitate management decisions.

One needs a large annotated image dataset with labeled diseased plant tissues to produce a precise model for automatic disease segmentation. The colossal effort of dataset annotation is ongoing, as highlighted in recent reviews on computer vision for scoring plant diseases (Singh et al., 2020; Abade et al., 2021; Li et al., 2021; Liu and Wang, 2021). Moreover, the international community in computer vision for plant pathology makes an additional effort by publicly sharing annotated datasets (Lu and Young, 2020). Notice that most available annotated datasets of diseased plants are in standard RGB color imaging and for disease stages when symptoms differ from healthy tissues quite well, by color and contrast. Several challenges exist for disease annotation in CFI at the earlier stages, such as (a) detection by the eye of the disease spots in noisy monochromatic images; (b) extremely tiny size of disease lesions; (c) a large number of scattered lesions to annotate. So far, to the best of our knowledge, only one annotated dataset of fluorescent images of diseased *Arabidopsis thaliana* plants is currently available (Pavicic et al., 2021). However, the automated annotation of *Botrytis cinerea* fungal disease was performed for disease severity exceeding 8%. Thus, the high human labor cost of manual annotation results in the lack of annotated fluorescence images of diseased plants suitable for disease segmentation (Lu and Young, 2020).

One way to circumvent this difficulty is to simulate a synthetic dataset with automated annotation. Using a synthetic dataset for the model training alleviates the annotation cost and augments the dataset, thus improving the model’s ability to generalize (Douarre et al., 2019; Abbas et al., 2021). This approach has been widely used in plant disease classification (Sun et al., 2020; Abbas et al., 2021; Cap et al., 2022). In contrast, in disease detection and segmentation, there are still only a few examples of the synthetic datasets (Douarre et al., 2019; Zhang et al., 2021). Additionally, since RGB cameras dominate plant disease monitoring (Iqbal et al., 2018), having the more affordable cost (Mavridou et al., 2019), image analysis methods are adapted for this type of imaging, which results in the creation of RGB synthetic datasets. However, RGB imaging is unsuitable for early disease diagnosis and is sensitive to illumination conditions that can significantly alter color-based segmentation accuracy (Iqbal et al., 2018; Mavridou et al., 2019).

To fill this gap, our goal was twofold: (1) to develop a novel methodology for generating fluorescent images of plants with an automated disease lesion annotation; (2) to illustrate the efficacy of the trained model on the synthetic dataset for segmentation lesions on an empirical CFI dataset of *Arabidopsis thaliana* infected by the bacterium *Pseudomonas syringae pv. tomato*. This traditional way of transfer learning from synthetic images to real ones is called the sim2real transfer. The first generation of sim2real on fluorescence imaging was proposed in 2019 on healthy *Arabidopsis thaliana* plants for leaf segmentation (Sapoukhina et al., 2019). Here, we extended this approach to the case of disease lesion segmentation. First, we analyzed the principal statistics of the empirical CFI dataset to derive some relationships that fluorescent synthetic data should respect. Second, we created a synthetic dataset under derived conditions. Third, we trained the U-Net model on synthetic data to segment disease lesions and transferred the model to the empirical CFI dataset. Finally, we discussed the conditions for successful sim2real transfer.

## 2 Materials and methods

### 2.1 Bacterial strain and culture conditions

A strain of *Pseudomonas syringae pv. tomato* (accession CFBP 7438) was obtained from CIRM-CFBP (INRAE Angers). This accession is a Rifampicin resistant variant strain obtained from strain DC3000. Bacteria were cultured on KB supplemented with Rifampicin (100 *μ*g/ml) to avoid any other contaminating bacteria. To produce the bacterial inoculum, we resuspended bacterial cells in sterile water to an OD600 = 0.5. Then, aliquots of the bacterial inoculum were diluted and plated on KB agar supplemented with Rifampicin to check that the concentration reached approximately 108 cfu.ml ^−1^.

### 2.2 Plant material and inoculation

After sowing, rosettes of *Arabidopsis thaliana* ecotype Col0 were grown on peat (Tray substrate Klasmann-Delimann France SARL, CS 71012, 38807 Bourgoin-Jallieu, France) and watered with fertilized water (N/P/K: 15/10/30, EC= 1.2 S). Plants were kept under short days conditions (photoperiod of 8h day and 16h night, the intensity of incident light was set to 150 *μ*E). The temperature was set at 21°C on days and 19°C during nights. Relative humidity was set to 60% during the development of rosettes (until three weeks after sowing), then raised to 95% after inoculation until the end of the experiment. Three weeks after sowing, plants were inoculated by spraying either sterile water (mock) or a bacterial suspension at OD600 = 0.5 and kept for 15days to develop disease symptoms.

To ensure that the observed symptoms were due to inoculation, the development of populations of strain DC3000 on *A. thaliana* Col0 was checked at the end of the experiment: mock and bacteria-inoculated rosettes were harvested and weighed. Total bacterial population sizes were quantified by macerating the rosettes in 10 ml of sterile water using a Stomacher 80 (Seward, London) for 2 min at maximum power. Every sample and appropriate dilutions were plated on KB supplemented with Rifampicin at 100 $\mu$g/ml. Therefore, we checked that populations of *Pseudomonas syringae* DC3000 reached 3.5 106 cfu.g ^−1^ of leaf tissues at the end of the experiment.

### 2.3 Chlorophyll fluorescence imaging and image acquisition

Visible disease symptoms do not provide complete information about plant health, and they are not the best indicator for estimating plant disease severity at the earlier stages of the infection. Plants experiencing biotic and abiotic stresses exhibit changes in chlorophyll fluorescence emission (Baker, 2008), which can be observed with fluorescence imaging.

One of the most widely studied parameters based on chlorophyll fluorescence is *F*_*v*_/*F*_*m*_ , also known as the maximum quantum efficiency of photosystem PSII (Baker, 2008). This parameter is calculated from *F*_*m*_ , the maximum fluorescence of a dark-adapted leaf, and *F*_*v*_ , the difference between *F*_*m*_ and the minimum fluorescence from a dark-adapted leaf, *F*_0_ . Up to now, the *F*_*v*_/*F*_*m*_=(*F*_*m*_−*F*_0_)/*F*_*m*_ parameter, has played an important role in plant stress research. It represents the maximum potential capacity of PSII reaction center, transforming the photon energy absorbed by PSII into photochemical energy. While non-stressed plants maintain a consistent *F*_*v*_/*F*_*m*_ value, various studies have shown that plants experiencing biotic or abiotic stresses have decreased *F*_*v*_/*F*_*m*_ values (Berger et al., 2006; Matous et al., 2006; Baker, 2008; Rolfe and Scholes, 2010; Wang et al., 2018), and changes in this parameter occur before visible disease symptoms occur (Rolfe and Scholes, 2010). Furthermore, healthy tissues were reported to yield *F*_*v*_/*F*_*m*_ values around 0.84 for numerous plant species (Maxwell and Johnson, 2000; Rousseau et al., 2013; Pavicic et al., 2021). Thus, using *F*_*v*_/*F*_*m*_ decrease as an indicator of plant stress, (Rousseau et al., 2013) developed a thresholding approach to segment diseased areas of bean leaflets infected by a common bacterial blight. The same approach was used by (Pavicic et al., 2021). The authors defined the pixel value threshold of *F*_*v*_/*F*_*m*_≤0.75 as the cutoff for symptomatic pixels of *Arabidopsis* leaflets infected with a *Botrytis* strain.

Since dark adaptation for *F*_*v*_/*F*_*m*_ measurements makes it difficult to translate to agricultural fields, other photosynthetic parameters and a combination of CFI with other imaging techniques have been studied for the detection of plant diseases (Bauriegel and Herppich, 2014; Mutka and Bart, 2015; Ivanov and Bernards, 2016). Despite its limitations, the *F*_*v*_/*F*_*m*_ measurement remains state-of-the-art for studying plant-pathogen interactions under laboratory conditions. In this study, we exploit the *F*_*v*_/*F*_*m*_ decay property to simulate diseased pixels on *Arabidopsis* plant images and thus create a synthetic dataset of fluorescent images of diseased plants.

*Real-Fluo-Healthy* and *Real-Fluo-Diseased* datasets described further were acquired with the PSI Open FluorCam FC 800-O (PSI, Brno, Czech Republic), capturing chlorophyll fluorescence images and estimating the maximum quantum yield of PSII, *F*_*v*_/*F*_*m*_ , on rosettes of *Arabidopsis thaliana* ecotype Col0. The system sensor was a CCD camera with a pixel resolution of 512 by 512 and a 12-bit dynamic range. The system included 4 LED panels divided into two pairs. One pair provided an orange actinic light with a wavelength of around 618 nm, with an intensity varying from 200 to 400 *μ*mol m^-2^ s^-1^. It provided a 2s pulse that allowed measuring the initial fluorescent state, *F*_0_ . The other pair provided a saturating pulse during 1s in blue wavelength, typically 455 nm, with an intensity of up to 3000 *μ*mol m ^−2^ s ^−1^ . The saturating pulse allowed us to collect the maximum fluorescence, *F*_*m*_ . CFI was used in a dark-adapted mode after a dark period of 45 min to produce maps with the fluorescent quantum efficiency *F*_*v*_/*F*_*m*_=(*F*_*m*_−*F*_0_)/*F*_*m*_ . We used a typical acquisition protocol, namely quenching analysis (Kolber et al., 1998), to measure *F*_0_ and *F*_*v*_/*F*_*m*_ parameters. First, the parameter *F*_0_ was measured using a modulated light of 0.1 *μ*mol m ^−2^ s ^−1^ . Then orange actinic light with intensities of 80 *μ*mol m ^−2^ s ^−1^ was used during the light-adapted period of 60 s. The illumination protocol also involved 6 pulses of 0.8 s duration of blue saturating light with an intensity of 1500 *μ*mol m ^−2^ s ^−1^ : 5 pulses during the light-adapted period and 1 pulse during the dark-relaxation period. Such an intensity of the saturating light pulse was chosen as it resulted in a ratio (*F*_*m*_−*F*_0_)/*F*_*m*_ of 0.82 measured on healthy plants and being close to the optimal value of 0.83 (Björkman and Demmig, 1987).

### 2.4 CVPPP dataset

To design plant shapes in *Synthetic-Fluo-Diseased* dataset, we used the open dataset provided in the Leaf Segmentation Challenge held as part of the computer vision problems in plant phenotyping *CVPPP* workshop (Minervini et al., 2016). *CVPPP* dataset consists in 27 RGB images of tobacco plants and 783 RGB images of *Arabidopsis* wild and mutant plants. We considered only the *Arabidopsis* dataset in this study. All images are hand-labeled to obtain ground truth masks for each leaf in the scene, as described in (Minervini et al., 2015). These masks are image files encoded in PNG where each segmented leaf is identified with a unique integer value, starting from 1, where 0 is the background. We used plant labels of *CVPPP* dataset to produce binary masks of plant shapes on which further we added texture of Gaussian noise simulating CFI.

### 2.5 Real-Fluo-Healthy dataset

To derive the principle measurements of CFI on healthy plants, we acquired 49 fluorescent images of *Arabidopsis* inoculated with sterile water (mock), named further as an empirical *Real-Fluo-Healthy* dataset. We emphasize that the dataset included only healthy plants.

Plants were imaged in chlorophyll fluorescence for 12 days after inoculation to obtain *F*_0_, *F*_*m*_ tiff images. We analyzed the distribution of the *F*_0_ and *F*_*m*_ parameters for *Real-Fluo-Healthy* images, deriving *μ*_*F*_0__, *σ*_*F*_0__, *μ*_*F*_*m*__, *σ*_*F*_*m*__ at each developmental stage (**Table 1**). These values were used further to simulate fluorescent quantum efficiency, *F*_*v*_/*F*_*m*_=(*F*_*m*_−*F*_0_)/*F*_*m*_, of healthy pixels of plants.

**Table 1:** Mean, *μ* , and standard deviation, *σ* , for chlorophyll fluorescence *F*_0_, *F*_*m*_ estimated on images from *Real-Fluo-Healthy* dataset at different dates after emergence of first leaves (cotyledons).

Time	*μ*_*F*_0__	*σ*_*F*_0__	*μ*_*F*_*m*__	*σ*_*F*_*m*__
Day 1	92.48	33.89	636.95	217.8
Day 5	104.84	33.24	711.33	200.27
Day 6	106.67	32.88	735.35	195.82
Day 7	101.21	31.11	735.91	192.7
Day 8	104.88	33.19	710.82	199.74
Day 11	107.99	29.46	785.74	183.04
Day 12	111.12	29.5	800.9	179.16

### 2.6 Real-Fluo-Diseased dataset

Four weeks after seedlings, *Arabidopsis thaliana* ecotype Col0 plants were inoculated with the bacterial *Pseudomonas syringae pv. tomato* strain DC3000 as described in Section 2.2. There were 18 dishes with four *Arabidopsis thaliana* plants per dish, inoculated with DC3000 bacterial strain. Each dish was imaged in chlorophyll fluorescence every two days after inoculation to obtain *F*_0_, *F*_*m*_, and *F*_*v*_/*F*_*m*_ tiff images. The dynamics of symptoms growth were followed till the eighth day after inoculation. It resulted in 108 images of size 512 x 512 containing four plants. The disease lesions were annotated with a thresholding approach based on the probability of misclassification of a healthy pixel into the class of diseased pixels (Rousseau et al., 2013). Thus, we created an empirical *Real-Fluo-Diseased* dataset used further to understand the effect of disease on the fluorescence parameter values. The dataset included only diseased plants.

Considering separately healthy (*h*) and diseased (*d*) pixels, we analyzed values of the mean and standard deviation of *F*_0_, *F*_*m*_ parameters for eight consecutive days (**Table 2**). As it was said before (Section 2.3), *F*_*v*_/*F*_*m*_ shows a significant decrease with aggravation of the disease symptoms. Thus, we calculated the differences between parameter values for healthy and diseased plant pixels as follows:


(1) $$\Delta_{\mu_{F_{0}}}=\frac{\mu_{F_{0}}^{\left(d\right)}-\mu_{F_{0\;}}^{\left(h\right)}}{\mu_{F_{0}}^{\left(h\right)}},\Delta_{\sigma_{F_{0}}}=\frac{\sigma_{F_{0}}^{\left(d\right)}-\sigma_{F_{0}}^{\left(h\right)}}{\sigma_{F_{0}}^{\left(h\right)}},\Delta_{\mu_{F_{m}}}=\frac{\mu_{F_{m}}^{\left(h\right)}-\mu_{F_{m}}^{\left(d\right)}}{\mu_{F_{m}}^{\left(h\right)}},\Delta_{\sigma_{F_{m}}}=\frac{\sigma_{F_{m}}^{\left(h\right)}-\sigma_{F_{m}}^{\left(d\right)}}{\sigma_{F_{m}}^{\left(h\right)}}$$

**Table 2:** Mean, *μ*, and standard deviation, *σ*, for chlorophyll fluorescence *F*_0_, *F*_*m*_ estimated on the healthy (*h*) and diseased (*d*) tissues of the plants over 18 dishes from *Real-Fluo-Diseased* dataset at different dates after the emergence of cotyledons.

Time	Healthy Pixels				Diseased Pixels			
	$\mu_{F_{0}}^{\left(h\right)}$	$\sigma_{F_{0}}^{\left(h\right)}$	$\mu_{F_{m}}^{\left(h\right)}$	$\sigma_{F_{m}}^{\left(h\right)}$	$\mu_{F_{0}}^{\left(d\right)}$	$\sigma_{F_{0}}^{\left(d\right)}$	$\mu_{F_{m}}^{\left(d\right)}$	$\sigma_{F_{m}}^{\left(d\right)}$
Day 0	62.86	15.25	418.49	88.84	56.08	18.36	257.26	89.57
Day 2	64.76	16.33	432.51	95.46	66.99	21.06	299.42	93.2
Day 5	67.17	16.79	438.92	96.36	74.87	21.67	302.2	89.98
Day 6	67.4	17.14	444.77	99.07	77.42	21.81	308.15	89.77
Day 7	68.79	17.4	447.74	99.96	75.1	22.18	305.9	91.56
Day 8	67.26	16.99	441.19	98.53	71.9	22.34	304.12	92.37

The found relationships among healthy and diseased pixels (1) allowed estimating the ranges for Δ_*i*_∈[*min*{Δ_*i*_}, *max*{Δ_*i*_}], where *i*∈{*μ*_*F*_0__, *σ*_*F*_0__, *μ*_*F*_*m*__, *σ*_*F*_*m*__} (**Table 3**). Further, we used these parameter variations induced by disease to generate a random set of parameters describing the fluorescence of diseased pixels. With parameter values obtained earlier in Section 2.5 for the healthy pixels, *μ*_*F*_0__,*σ*_*F*_0__,*μ*_*F*_*m*__,*σ*_*F*_*m*__ , we obtain:


(2) $$\mu_{F_{0}}^{\left(r\right)}=\mu_{F_{0}}\left(1+{\text{Δ}}_{\mu_{F_{0}}}^{\left(r\right)}\right),\;\mu_{F_{m}}^{\left(r\right)}=\mu_{F_{m}}\left(1-{\text{Δ}}_{\mu_{F_{m}}}^{\left(r\right)}\right),\;\sigma_{F_{0}}^{\left(r\right)}=\sigma_{F_{0}}\left(1+{\text{Δ}}_{\sigma_{F_{0}}}^{\left(r\right)}\right),\;\sigma_{F_{m}}^{\left(r\right)}=\sigma_{F_{m}}\left(1-{\text{Δ}}_{\sigma_{F_{m}}}^{\left(r\right)}\right),$$

**Table 3:** Estimated ranges for Δ_i_, *i*∈{*μ*_*F*_0__, *σ*_*F*_0__, *μ*_*F*_*m*__, *σ*_*F*_*m*__} for diseased pixels.

	Δ_*μ*_*F*_0___	Δ_*σ*_*F*_0___	Δ_*μ*_*F*_*m*___	Δ_*σ*_*F*_*m*___
*min*	0.07	0.27	0.3	0.06
*max*	0.12	0.32	0.32	0.09

where a random (*r*) value of ${\text{Δ}}_{i}^{\left(r\right)}\in \left[min\left\{{\text{Δ}}_{i}\right\},max\left\{{\text{Δ}}_{i}\right\}\right]$ , *i*∈{*μ*_*F*_0__, *σ*_*F*_0__, *μ*_*F*_*m*__, *σ*_*F*_*m*__} .

To analyze disease symptoms growth, we calculated severity, *S* , and the plant size, *P* :


(3) $$S=\frac{d}{H*W}*100\%,$$

(4) $$P=\frac{p}{H*W}*100\%,$$

where *d* is the number of diseased pixels, *p* is the number of plant pixels, *H* and *W* are the image’s height and width, respectively.

To create a dataset for model testing, we used *Real-Fluo-Diseased* dataset. We divided every original image with four plants into four resized 128 x 128 images that were finally added to the dataset. Thus, we obtained 432 fluorescent images of diseased *Arabidopsis thaliana* plants.

### 2.7 Synthetic-Fluo-Diseased dataset

**Figure 1:**
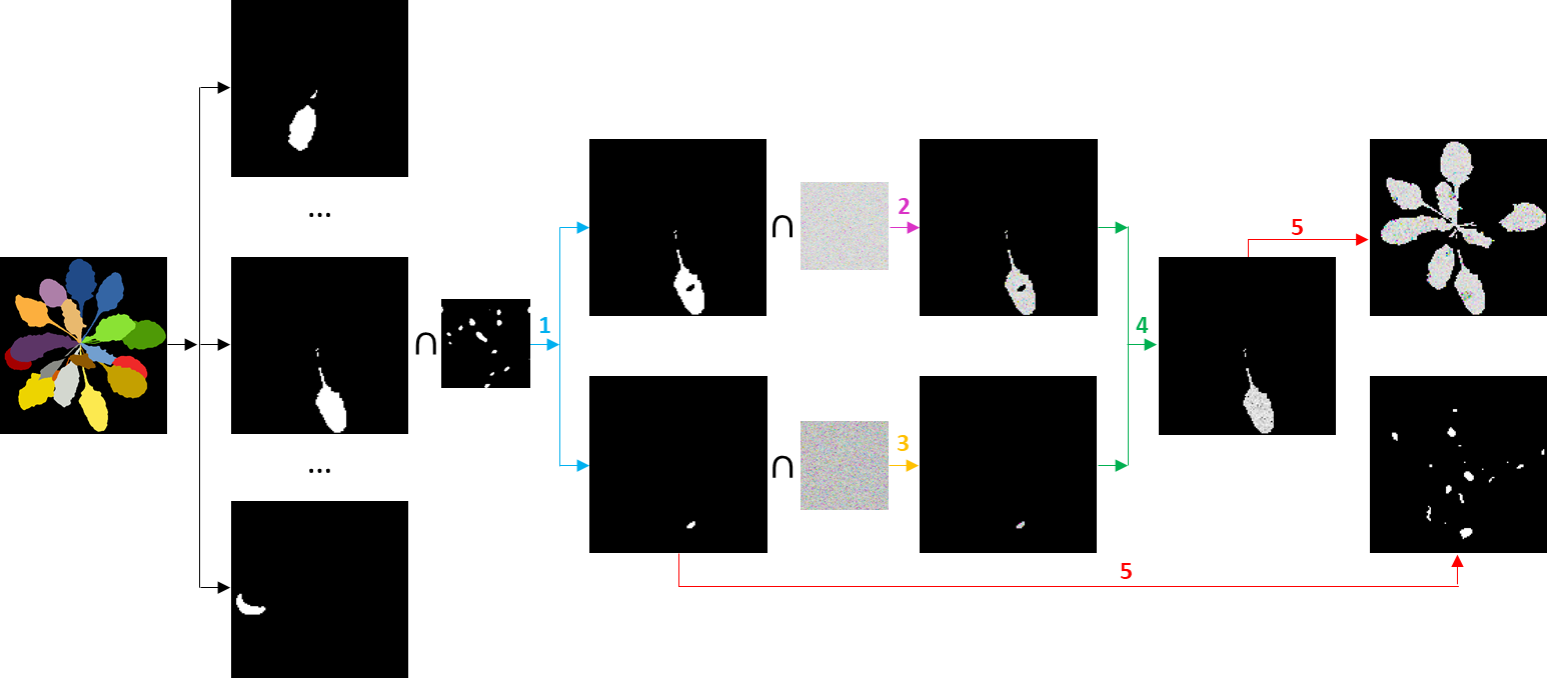
Algorithm generating a synthetic fluorescent diseased plant and its lesion annotation. Iterations over the leaves with steps 1 - 5 are described in the text.

We used plant labels of *CVPPP* dataset to produce binary masks of leaves for every *Arabidopsis* image as it is shown in **Figure 1**. In *CVPPP* labels, every leaf was determined by its consistent color. So we used getcolor() function from Python Imaging Library (1.1.7 version) to derive the list of colors used to depict leaves separately. Then for every color from the list, we selected pixels belonging to the same leaf and created its binary mask. The resulting leaf masks of size 128 x 128 pixels were then used to simulate fluorescence images of *Arabidopsis* plants.

For every leaf, we performed the following steps:

1. We produced two binary masks: a lesions mask using a speckle algorithm (Goodman, 2007) and a healthy leaf mask.

2. We produced a noisy field, *x* ^(*h*)^ , with randomly sampled parameters (*μ*_*F*_0__, *σ*_*F*_0__) from **Table 1**, learned earlier from the *Real-Fluo-Healthy* dataset:


(5) $$x^{\left(h\right)}=1-\frac{N\left(\mu_{F_{0}},\sigma_{F_{0}}^{2}\right)}{N\left(\mu_{F_{m}},\sigma_{F_{m}}^{2}\right)},$$

where $N\left(\mu_{i},\sigma_{i}^{2}\right)$ is a Gaussian noise realization. Using the mask for healthy leaf tissue, we cut from the noisy field, *x* ^(*h*)^ , a synthetic fluorescent healthy leaf part.

3. Using expressions (2), we generated a random set of parameters, $\left\{\mu_{F_{0}}^{\left(r\right)},\mu_{F_{m}}^{\left(r\right)},\sigma_{F_{0}}^{\left(r\right)},\sigma_{F_{m}}^{\left(r\right)}\right\}$ , that produced a noisy field, *x* ^(*d*)^, describing diseased pixels:


(6) $$x^{\left(d\right)}=1-\frac{N\left(\mu_{F_{0}}^{\left(r\right)},\sigma_{F_{0}}^{{\left(r\right)}^{2}}\right)}{N\left(\mu_{F_{m}}^{\left(r\right)},\sigma_{F_{m}}^{{\left(r\right)}^{2}}\right)},$$

where $N\left(\mu_{i},\sigma_{i}^{2}\right)$ is a Gaussian noise realization. From the noisy field, *x* ^(*d*)^ , we cut synthetic fluorescent lesions by applying the binary lesion mask.

4. We assembled healthy and diseased parts into a synthetic fluorescent diseased leaf.

5. We added the resulting simulated fluorescent leaf to the other leaves obtained earlier. We copied the corresponding lesions mask to the GT binary mask of the disease spread.

Repeating these five steps, we obtained a synthetic fluorescent diseased plant and an automatic annotation of disease lesions (**Figure 1**).

To augment the number of images in the synthetic dataset, we produced *n* synthetic fluorescent examples for every GT label from *CVPPP* dataset. The resulting *Synthetic-Fluo-Diseased* dataset included only diseased *Arabidopsis thaliana* plants.

### 2.8 U-Net model

Most computer vision models treating images of diseased plants focus on disease classification or disease detection and not on segmentation of plant diseased tissues (Abade et al., 2021). Moreover, the predominance of RGB imaging significantly affected the development of color-based semantic segmentation approaches. Among deep learning architectures used for the segmentation of disease spots in RGB images, PSP Net, U-Net, and DeepLab v3+, or transformer-based architecture, could be considered as the most suitable for monochromatic CFI. Here, since we did not focus on providing a new architecture nor claim any optimality on the choice of a model, we used a standard U-Net model (Ronneberger et al., 2015) for segmentation of disease lesions on the *Arabidopsis*plants.

The segmentation of the lesions was considered to be a pixel-wise classification where the pixel of the lesion should be detected among the other pixels of the image. Each pixel was therefore classified among three mutually exclusive classes: healthy pixels, diseased pixels, and background. It means that a three-component one-hot vector labeled every pixel.

**Figure 2:**
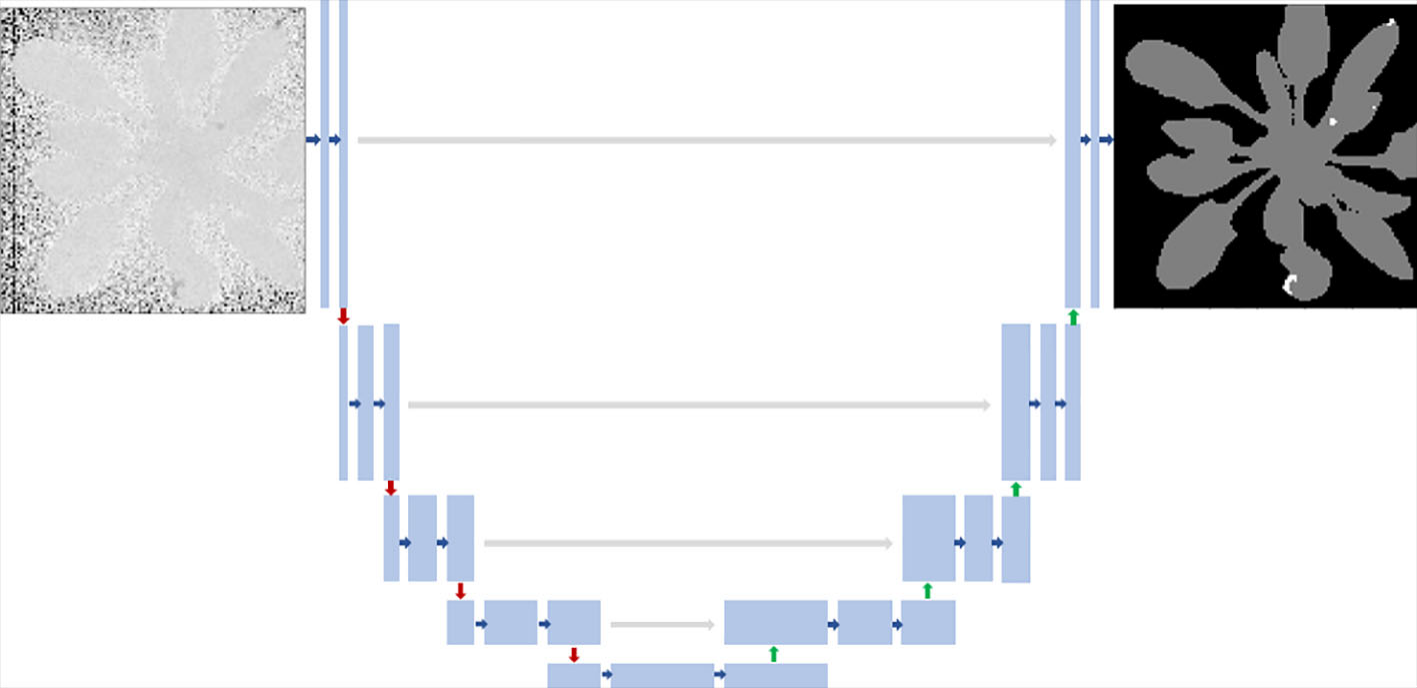
U-Net architecture. Each blue box corresponds to a multi-channel feature map. Blue arrows indicate convolution layers. Gray arrows indicate the merging of the context and localization information done by concatenating the features from the contracting path with the corresponding ones in the expansion path. Red arrows indicate max-pooling. Green arrows mean upsampling. Input fluorescent image had 128x128 pixels, and the model’s output was a three-channel binary image: healthy pixels, diseased pixels, and the background.

The U-Net model (Ronneberger et al., 2015) was used originally for the pixel-wise classification of biomedical images (2). U-Net architecture is divided into the contracting/downsampling path, the bottleneck, and the expanding/upsampling path. The encoder-decoder type architecture with skipped connections combines low-level feature maps with higher-level ones and enables precise pixel classification. A large number of feature channels in upsampling part allows propagating context information to higher resolution layers. The model’s output was a three-channel label that indicated the class of every pixel, as shown in **Figure 2**.

All activation functions in the convolutional layers were rectified linear units, ReLU (He et al., 2015). The last layer before the prediction was a softmax activation with three classes. Images and labels from all datasets were resized to 128 x 128 pixels. Using ground truth (GT) labels, we created three-channel labels. To reinforce the learning of the lesion class, which was highly unbalanced, we replaced the encoder with an InceptionV3 backbone pre-trained on ImageNet (Yakubovskiy, 2019). The decoder was not changed from the original description (Ronneberger et al., 2015). We empirically found that the best performances were obtained when all skipped connections were kept, in accordance with the intrinsic multiscale nature of plants (Rousseau et al., 2015b).

### 2.9 Model training

In addition to the data augmentation techniques used to simulate fluorescent images from *CVPPP* RGB dataset (Section 2.7), we applied a standard data augmentation strategy to reduce overfitting and improve model generalization. For this data augmentation, we used Albumentations library (Buslaev et al., 2020). While the data augmentation strategies of Section 2.7 focused on contrast and noise distribution, here we generated geometrical transformations such as horizontal flip, vertical flip, and random rotation at 90 degrees and applied them to a 0.7 shuffled training dataset. The values of three transformations were all randomly selected from their range. Moreover, when an image was transformed, its annotation image was transformed similarly. In addition, since the transformation parameters were randomly selected, it was necessary to generate a random number. To ensure the generated data is the same in each epoch during the training process, we fixed the value of the random seed as 42. As a result, 9317 training data pairs were generated from 5481 training samples and transformation methods.

It was shown that for high levels of imbalance, loss functions based on overlap measures appeared to be more robust (Sudre et al., 2017). Through all of our experiments, we minimized dice loss:


(7) $$D\left(y,y^{*}\right)=\frac{2\sum_{ij}y_{ij}y_{ij}^{*}+\epsilon }{\sum_{ij}y_{ij}+\sum_{ij}y_{ij}^{*}+\epsilon },$$

where *y* is a model prediction with values *y*_*ij*_ , *y* ^*^ is a ground truth label with values $y_{ij}^{*}$ and *ϵ*=0.001 is used here to ensure the coefficient stability by avoiding the numerical issue of dividing by 0.

In the optimization process, the Adam method was applied with the learning rate *l*_*r*_=0.003 , and the other parameters were consistent with those in the original manuscript (Kingma and Ba, 2014). Our training procedure consisted of splitting the data into 80% and 20% training and cross-validation respectively. We shuffled the dataset examples at the beginning of each epoch and used a batch size of 16 examples. We also implemented batch normalization before each activation. The hardware used for training the model was a GPU server equipped with an Intel Core i9-9900K CPU and an NVIDIA TITAN V GPU. We implemented our model with a high-level neural network API called Keras (Chollet et al., 2015) with the 241 Tensorflow (Abadi et al., 2015) backbend running on the Ubuntu 18.03 operating system.

### 2.10 Model testing

To verify the model’s performance, we used a Test dataset including *Real-Fluo-Diseased* images (Section 2.6). To assess the quality of pixel-wise segmentation, we computed (1) True Positives (*TP* ): the number of diseased pixels present in both prediction and GT mask; (2) False Positives (*FP* ): the number of diseased pixels present in prediction but absent in GT mask; (3) False Negatives (*FN* ): the number of diseased pixels absent in prediction but present in GT mask. Knowing these numbers, we can estimate *Recall*:


(8) $$R=\frac{TP}{TP+FN},$$

that describes the fraction of correctly classified diseased pixels compared to the total number of diseased pixels in GT mask. Moreover, *Precision* value:


(9) $$P=\frac{TP}{TP+FP},$$

gives us the fraction of correctly classified diseased pixels among all predicted diseased pixels. And finally, *F*1 -score gives us a global assessment of the model performance:


(10) $$F1\text{−score}=\frac{TP}{TP+0.5\left(FP+FN\right)}.$$

The formula for the standard *F*1-score is the harmonic mean of the *Precision* and *Recall*. A perfect model has an *F*1 -score of 1.

### 2.11 Model training strategies

For each experiment, we changed the dataset composition (synthetic or empirical images) for learning, fine-tuning, or testing. Then, we applied a standard scheme of sim2real transfer:

**A** *Train: empirical. Test: empirical.*

It was a reference to see what the model could learn from a small empirical dataset of fluorescent plant images. At this stage, the analysis of model errors allowed us to choose the pre-processing strategy for forming the Test dataset from *Real-Fluo-Diseased* images.

**A** *Train: empirical. Test: empirical pre-processed.*

It was a reference to see how much model precision we gained using the chosen pre-processing strategy.

**B** *Train: synthetic. Test: empirical pre-processed.*

At this stage, we performed the model training on *Synthetic-Fluo-Diseased* dataset and tested the model on the pre-processed *Real-Fluo-Diseased*. This training strategy validated the quality and relevance of the created *Synthetic-Fluo-Diseased* dataset. Furthermore, the error analysis of the model allowed us to establish the criteria for sim2real transfer strategy described below.

**C** *Train: synthetic + n pre-processed empirical examples. Test: n pre-processed empirical examples.*

Here, we used the first sim2real transfer strategy. It consisted of injection of some empirical images from *Real-Fluo-Diseased* for which the model failed to correctly segment disease lesion into the *Synthetic-Fluo-Diseased* dataset and training the model from scratch on this mixed dataset. Then, we tested the model on the other *Real-Fluo-Diseased* images not used for the training.

**D** *Train: synthetic. Fine-tune: n pre-processed empirical Test: n pre-processed empirical examples.*

We applied the second sim2real transfer strategy to make the model recognize better the contrast between diseased and healthy pixels. This strategy consists of the model pre-training on the *Synthetic-Fluo-Diseased* dataset and further fine-tuning the model classifier layer on some empirical examples. Further, we tested the model on the not-seen examples from the *Real-Fluo-Diseased* dataset.

Note that strategies (A-D) used only images of diseased plants.

## 3 Results

### 3.1 Pre-processing strategy for a Test dataset

The model training and testing on *Real-Fluo-Diseased* dataset (Section 2.11, strategy A) revealed that the model failed to segment disease lesions correctly if lesions looked like scattered corrupted pixels and severity was less than 0.24%. The majority of such images were from the first post-inoculation day, when disease severity varied from 0.012% to 0.24%, with a mean of 0.055%. Several studies showed that in pre-symptomatic detection of plant diseases, differences between healthy and diseased plant tissues become statistically significant from the second day after inoculation (Boureau et al., 2002; Grishina et al., 2021). Thus, we proposed to pre-process the dataset in two steps: 1) we applied morphological operations to eliminate corrupted pixels and to uniform lesions if they included small holes; 2) we eliminated images with *S*≤0.24*%* . As a result, our final Test dataset contained 153 examples with a severity mean of 1%.

### 3.2 Principle statistics of Real-Fluo-Healthy dataset, Real-Fluo-Diseased and Synthetic-Fluo-Diseased datasets

**Figure 3:**
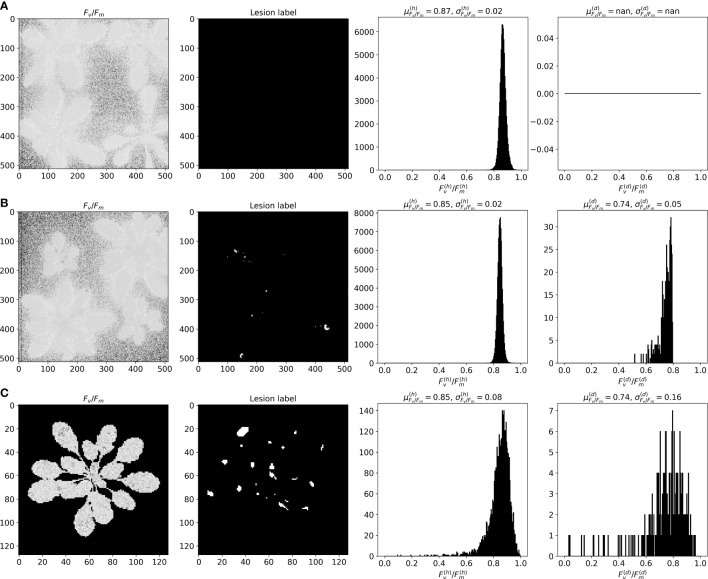
Fluorescent image examples from **(A)** *Real-Fluo-Healthy*, **(B)** *Real-Fluo-Diseased* and **(C)** *Synthetic-Fluo-Diseased* datasets with corresponding *F*_*v*_/*F*_*m*_ histograms for healthy (*h*) and diseased (*d*) pixels.

The resulting mean value and standard deviation of the both chlorophyll fluorescence parameters *F*_0_ and *F*_*m*_ for *Real-Fluo-Healthy* dataset are given in **Table 1**. The order of magnitude of the mean value and standard deviation of *F*_0_ and *F*_*m*_ remained in the same range in our experiment. **Figure 3A** shows a *Real-Fluo-Healthy* image example of the maximum quantum yield *F*_*v*_/*F*_*m*_ and its values distribution.

**Figure 4:**
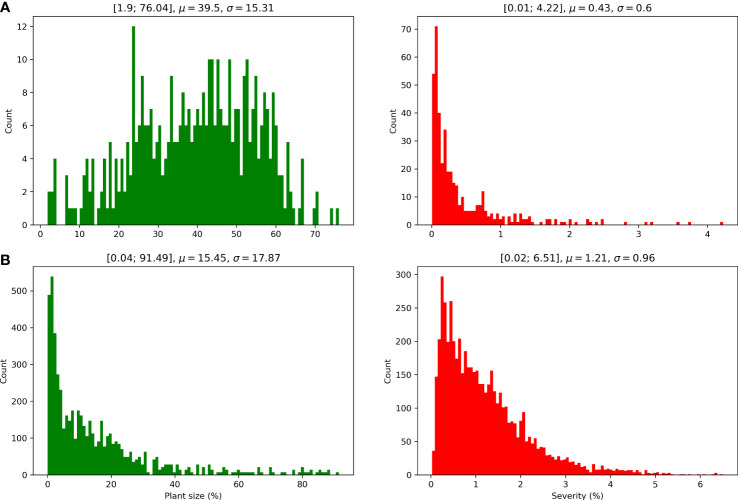
Plant size and severity distributions of **(A)** *Real-Fluo-Diseased* and **(B)** *Synthetic-Fluo-Diseased* datasets.

As shown in **Figure 3B**, *Real-Fluo-Diseased* dataset has disease symptoms of extremely small size. The mean disease severity of the dataset equals 0.42%, and maximum severity - 4.2% (**Figure 4A**). The plants of *Real-Fluo-Diseased* dataset have the mean size of 39.5%, some images have minimum plant size of 1.9% (**Figure 4A**). **Figure 3B** shows that for the considered maximum quantum yield *F*_*v*_/*F*_*m*_ image of a diseased plant, the mean values of *F*_*v*_/*F*_*m*_ are higher for the healthy pixels than for diseased ones. **Table 2** confirms this state.

Analyzing values of *F*_0_ and *F*_*m*_ parameters obtained separately for healthy and diseased pixels (**Table 2**), we concluded that:

• For healthy and diseased pixels, mean and standard deviation values of *F*_0_ are less than *F*_*m*_ values:


$$\mu_{F_{0}}^{\left(h\right)}<\mu_{F_{m}}^{\left(h\right)},\sigma_{F_{0}}^{\left(h\right)}<\sigma_{F_{m}}^{\left(h\right)},\mu_{F_{0}}^{\left(d\right)}<\mu_{F_{m}}^{\left(d\right)},\sigma_{F_{0}}^{\left(d\right)}<\sigma_{F_{m}}^{\left(d\right)}.$$

• The presence of disease lesions on the leaves increases *μ*_*F*_0__ , *σ*_*F*_0__ and decreases *μ*_*F*_*m*__, *σ*_*F*_*m*__ . It gives:


$$\mu_{F_{0}}^{\left(h\right)}<\mu_{F_{0}}^{\left(d\right)},\sigma_{F_{0}}^{\left(h\right)}<\sigma_{F_{0}}^{\left(d\right)},\mu_{F_{m}}^{\left(d\right)}<\mu_{F_{m}}^{\left(h\right)},\sigma_{F_{m}}^{\left(d\right)}<\sigma_{F_{m}}^{\left(h\right)}.$$

• Consequently, $\mu_{F_{v}/F_{m}}^{\left(d\right)}<\mu_{F_{v}/F_{m}}^{\left(h\right)}$ , $\sigma_{F_{v}/F_{m}}^{\left(h\right)}<\sigma_{F_{v}/F_{m}}^{\left(d\right)}$ . Note that this conclusion about the relationship between *F*_*v*_/*F*_*m*_ for healthy and diseased pixels is consistent with previous studies (Maxwell and Johnson, 2000; Matous et al., 2006; Baker, 2008; Rousseau et al., 2013; Pavicic et al., 2021).

**Figure 5:**
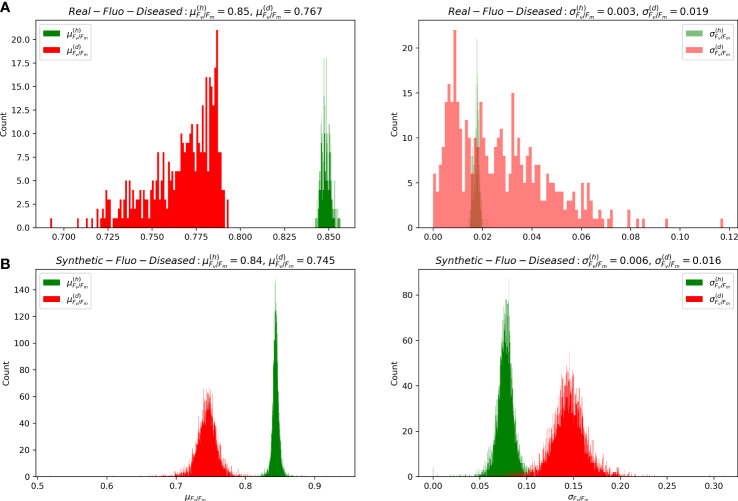
Distributions of mean and standard deviation of *F*_*v*_/*F*_*m*_ for healthy (*h*) and diseased (*d*) pixels of images from **(A)** *Real-Fluo-Diseased* and **(B)** *Synthetic-Fluo-Diseased* datasets.

The obtained ranges for Δ_*i*_∈*ℝ* values (1), where *i*∈{*μ*_*F*_0__, *σ*_*F*_0__, *μ*_*F*_*m*__,*σ*_*F*_*m*__} , are summarized in **Table 3**:

As can be seen from the **Figures 3B, C**, that distributions of mean and standard deviations of *F*_*v*_/*F*_*m*_ for considered images from *Synthetic-Fluo-Diseased* and *Real-Fluo-Diseased* differ. It is not the only difference between synthetic and empirical datasets. **Figure 4** shows that plants on the images from *Synthetic-Fluo-Diseased* are smaller than in *Real-Fluo-Diseased* dataset, while the disease severity is higher. The small plant size came from the *CVPPP* dataset used to simulate plant shapes for the synthetic images. We kept such a difference in plant size to make the trained model more general and to be able to recognize disease lesions even on tiny plants. If we compare the values of *F*_*v*_/*F*_*m*_ over images in both datasets (**Figure 5**), we will see that *Synthetic-Fluo-Diseased* and *Real-Fluo-Diseased* datasets differ a lot. **Figure 5A** shows that in *Real-Fluo-Diseased* datasets, *F*_*v*_/*F*_*m*_ mean values of healthy and diseased pixels does not overlap while in *Synthetic-Fluo-Diseased* their do overlap. The values of standard deviations of *F*_*v*_/*F*_*m*_ is higher for *Synthetic-Fluo-Diseased* in comparison with *Real-Fluo-Diseased*.

### 3.3 Segmentation scores for the dataset augmentation strategies

**Table 4** shows the model performance results tested under conditions of the defined earlier training strategies. The model training/testing on empirical fluorescent images showed that the model could distinguish lesion pixels from healthy ones. Moreover, the model’s error analysis showed that the model had difficulties segmenting both the very tiny disease lesions, with overall severity of less than 0.24%, and the lesions with corrupted pixels. Thus, we developed a two-stage pre-processing strategy for the Test dataset described in Section 3.1. The second line in the **Table 4** shows the model performance 100% gain for the pre-processed Test dataset. Thus, we validated the pre-processing strategy and applied it to training strategies (B-D).

**Table 4:** Performance metrics on test samples for various training strategies (Section 2.11).

Training strategy	*Precision*	*Re call*	*F*1-score
**A** *Train: emp. (530). Test: emp. (107)*	0.448	0.306	0.347
**A** *Train: emp. (530). Test: emp.-proc. (38)*	0.800	0.685	0.725
**B** *Train: synth. (9317). Test: emp.-proc. (153)*	0.723	0.793	0.744
**C** *Train: synth. + 73 emp.-proc. (9441). Test: emp.-proc. (133)*	0.813	0.648	0.766
**D** *Train: synth. (9317). Fine-tune: 73 emp.-proc. Test: emp.-proc. (133)*	0.853	0.648	0.752

The number of training examples in brackets is given after applying a standard data augmentation procedure.

**Table 4** shows that the model trained on the synthetic data recognizes disease lesions quite well on the empirical fluorescent images. Therefore, we can conclude that the developed approach of simulating CFI captures the principle features of fluorescent images of diseased plants. The analysis of the model errors showed that the lesion segmentation with *F*1−score<0.7*%* corresponds to images with $\mu_{F_{v}/F_{m}}^{\left(d\right)}>0.76$ . If we compare distributions of $\mu_{F_{v}/F_{m}}^{\left(d\right)}$ for synthetic and empirical images in **Figure 5**, we will see that there is a lack of images with $\mu_{F_{v}/F_{m}}^{\left(d\right)}>0.76$ in the *Synthetic-Fluo-Diseased* dataset. Thus, we decided to inject 73 empirical images into the synthetic dataset, that is, a quarter of the images with $\mu_{F_{v}/F_{m}}^{\left(d\right)}>0.76$ , to test a sim2real transfer strategies (C, D).

**Figure 6:**
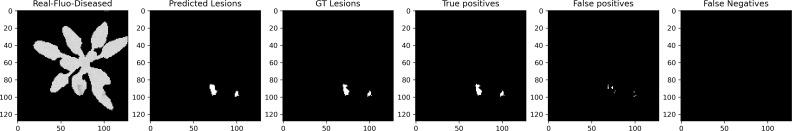
The best result of lesion segmentation on pre-processed *Real-Fluo-Diseased* with the model trained on the *Synthetic-Fluo-Diseased* dataset. *Precision* = 0.982, *Recall* = 1.000, *F*1-score = 0.925.

The presence of 73 empirical fluorescent images in the Synthetic dataset slightly improved the *F*1-score. **Figure 6** illustrates the best model result of lesion segmentation on Real-Fluo-Diseased images. It means that our criteria for sim2real transfer were chosen right. However, the number of empirical images should be increased to give a more pronounced gain of the model precision in lesion segmentation. The results of the model fine-tuning are close to the results of the model training from scratch. Thus, both sim2real transfer strategies can be applied to improve the model’s precision.

## Discussion

In this paper, we proposed a novel methodology to generate fluorescent images of diseased *Arabidopsis* plants with an automated lesion annotation. Using the U-Net neural network, we demonstrated that created synthetic dataset could be successfully used for disease segmentation on empiric fluorescent plant images at the early disease stages. Creating and using such synthetic data can be a powerful technique to facilitate the application of deep learning methods in precision crop protection.

Our method of generating fluorescent synthetic images is an essential contribution to the availability of various datasets in digital plant phenotyping, which serve to verify models’ robustness and generalization. Despite some synthetic and empirical set otherness, the U-Net model, trained on the synthetic data, showed 0.793% recall and 0.723% average precision for disease segmentation on the empirical fluorescent dataset. One could try to improve this segmentation score by using other neural network architectures, for example, an improved DeepLab v3+ (Yuan et al., 2022). Also important to note that we did not use domain adaptation between the empirical and synthetic datasets. Such compensation for the possible discrepancy between the two domains could be investigated in the latent space of the segmentation algorithm. It would constitute a viable way to increase model performance.

We demonstrated that the extremely tiny size of disease lesions on the first post-inoculation day was the main difficulty for precise lesion segmentation. The low model performance could indicate that the *F*_*v*_/*F*_*m*_ ratio is not a good indicator of diseases symptoms at such an early infection stage. Indeed, a general drawback of the *F*_*v*_/*F*_*m*_ parameter is the lack of specificity as it is influenced by many similar abiotic or biotic stress factors (Belin et al., 2013; Kalaji et al., 2017; Kim et al., 2019; Pérez-Bueno et al., 2019; Valcke, 2021; Zhang et al., 2022). This can be overcome by combining several CFI parameters (Méline et al., 2020) or by combining CFI with other imaging techniques (Bauriegel and Herppich, 2014; Wang et al., 2018; Pavicic et al., 2021). The U-Net model failed to segment disease lesions with overall disease severity of less than 0.24%. Usually, studies of automated disease quantification operate with plants at later disease stages, when minimum severity exceeds 8% (Lin et al., 2019; Pavicic et al., 2021; Yuan et al., 2022). At these stages, CFI imagining techniques allow estimating disease severity with a thresholding approach, using the *F*_*v*_/*F*_*m*_ threshold (≤0.75) to consider a pixel as diseased (Rousseau et al., 2013; Pavicic et al., 2021).

Our approach is generic and applicable to any crop after obtaining an accurate estimation of the *F*_*v*_/*F*_*m*_ statistics on the diseased and healthy parts of the plants. Currently, some technical challenges of CFI, such as dark adaptation for *F*_*v*_/*F*_*m*_ measurements, make it difficult to translate to agricultural fields. However, some studies illustrate the application of CFI to detect fungal diseases under field conditions (Bauriegel and Herppich, 2014; Ivanov and Bernards, 2016). New light-adapted chlorophyll fluorescence parameters were developed and used (Ivanov and Bernards, 2016). Moreover, combining with the other imaging technologies is another means for the wide-scale use of CFI (Bauriegel and Herppich, 2014; Wang et al., 2018).

The use of a synthetic dataset was investigated here with fluorescence imaging. Other imaging modalities are commonly used to monitor plant-pathogen interactions. This includes passive or active thermography, multispectral reflectance imaging in various wavelength ranges, and coherent speckle imaging (Mohammad-Razdari et al., 2022). It would be interesting to revisit and extend the approach of this paper to these plant imaging modalities. The physics of each of them differs from fluorescence imaging. Therefore the image production model would have to be adapted. The rest of the statistical methodology proposed in this article to train neural networks on synthetic datasets would remain unchanged.

In conclusion, the proposed method of generating fluorescent images of diseased plants makes a valuable tool for deepening our understanding of host-pathogen interactions and thus facilitating the development of durable resistance strategies. Moreover, it can contribute to developing highly efficient models for segmenting disease lesions that can be used for intelligent crop treatment technologies, reducing the amount of sprayed fungicides.

## Data availability statement

The raw data supporting the conclusions of this article will be made available by the authors, without undue reservation.

## Author contributions

NS analyzed datasets, coded both a generator of *Synthetic-Fluo-Diseased* dataset and model training/testing, analyzed simulation results, and wrote the article. TB provided *Real-Fluo-Healthy* and *Real-Fluo-Diseased* datasets and participated in article writing. DR conceived the idea of the synthetic dataset, proposed the method, supervised the whole project, and conducted article writing. All authors have read and approved the final manuscript.

## Acknowledgments

Authors gratefully acknowledge the PHENOTIC platform node https://doi.org/10.15454/U2BWFJ of the PHENOME National French infrastructure of plant phenotyping. This work was supported by the French National Research Agency (ANR), the Investments for the Future program (PIA), - the project PHENOME, ANR-11-INBS-0012.

## Conflict of interest

The authors declare that the research was conducted in the absence of any commercial or financial relationships that could be construed as a potential conflict of interest.

## Publisher’s note

All claims expressed in this article are solely those of the authors and do not necessarily represent those of their affiliated organizations, or those of the publisher, the editors and the reviewers. Any product that may be evaluated in this article, or claim that may be made by its manufacturer, is not guaranteed or endorsed by the publisher.

## References

[B1] Abade A. Ferreira P. A. de Barros Vidal F. (2021). Plant diseases recognition on images using convolutional neural networks: A systematic review. Comput. Electron. Agric. 185, 106125. doi: 10.1016/j.compag.2021.106125

[B2] Abadi M. Agarwal A. Barham P. Brevdo E. Chen Z. Citro C. . (2015). TensorFlow: large-scale machine learning on heterogeneous systems.

[B3] Abbas A. Jain S. Gour M. Vankudothu S. (2021). Tomato plant disease detection using transfer learning with c-GAN synthetic images. Comput. Electron. Agric. 187, 106279. doi: 10.1016/j.compag.2021.106279

[B4] Ampatzidis Y. (2018). Applications of artificial intelligence for precision agriculture: Ae529, 12/2018. EDIS 2018. doi: 10.32473/edis-ae529-2018

[B5] Baker N. R. (2008). Chlorophyll fluorescence: A probe of photosynthesis *in vivo* . Annu. Rev. Plant Biol. 59, 89–113. doi: 10.1146/annurev.arplant.59.032607.092759 18444897

[B6] Bauriegel E. Herppich W. B. (2014). Hyperspectral and chlorophyll fluorescence imaging for early detection of plant diseases, with special reference to fusarium spec. infections on wheat. Agriculture 4, 32–57. doi: 10.3390/agriculture4010032

[B7] Belin É. Rousseau D. Boureau T. Caffier V. (2013). Thermography versus chlorophyll fluorescence imaging for detection and quantification of apple scab. Comput. Electron. Agric. 90, 159–163. doi: 10.1016/j.compag.2012.09.014

[B8] Berger S. Benediktyová Z. Matouš K. Bonfig K. Mueller M. J. Nedbal L. . (2006). Visualization of dynamics of plant–pathogen interaction by novel combination of chlorophyll fluorescence imaging and statistical analysis: differential effects of virulent and avirulent strains of p. syringae and of oxylipins on *A. thaliana* . J. Exp. Bot. 58, 797–806. doi: 10.1093/jxb/erl208 17138624

[B9] Björkman O. Demmig B. (1987). Photon yield of O2 evolution and chlorophyll fluorescence characteristics at 77 K among vascular plants of diverse origins. Planta 170, 489–504. doi: 10.1007/BF00402983 24233012

[B10] Boureau T. Routtu J. Roine E. Taira S. Romantschuk M. (2002). Localization of *hrpA*-induced *Pseudomonas syringae* pv. tomato dc3000 in infected tomato leaves. Mol. Plant Pathol. 3, 451–460. doi: 10.1046/j.1364-3703.2002.00139.x 20569352

[B11] Buslaev A. Parinov A. Khvedchenya E. Iglovikov V. I. Kalinin A. A. (2020). Albumentations: Fast and flexible image augmentations. Information 11, 125. doi: 10.3390/info11020125

[B12] Cap Q. H. Uga H. Kagiwada S. Iyatomi H. (2022). LeafGAN: An effective data augmentation method for practical plant disease diagnosis. IEEE Transactions on Automation Science and Engineering 19, 1258–1267. doi: 10.1109/TASE.2020.3041499

[B13] Chollet F. . (2015) Keras. Available at: https://keras.io.

[B14] Douarre C. Crispim-Junior C. F. Gelibert A. Tougne L. Rousseau D. (2019). Novel data augmentation strategies to boost supervised segmentation of plant disease. Comput. Electron. Agric. 165, 104967. doi: 10.1016/j.compag.2019.104967

[B15] Goodman J. W. (2007). Speckle phenomena in optics: Theory and applications (Bellingham, Washington 440 USA: SPIE Press).

[B16] Grishina A. Sherstneva O. Grinberg M. Zdobnova T. Ageyeva M. Khlopkov A. . (2021). Pre-symptomatic detection of viral infection in tobacco leaves using pam fluorometry. Plants 10, 2782. doi: 10.3390/plants10122782 34961253 PMC8707847

[B17] He K. Zhang X. Ren S. Sun J. (2015). Delving deep into rectifiers: Surpassing human-level performance on imagenet classification. In Proceedings of the IEEE international conference on computer vision. 1026–1034. doi: 10.48550/arXiv.1502.01852

[B18] Iqbal Z. Khan M. A. Sharif M. Shah J. H. Rehman M. H. U. Javed K. (2018). An automated detection and classification of citrus plant diseases using image processing techniques: A review. Comput. Electron. Agric. 153, 12–32. doi: 10.1016/j.compag.2018.07.032

[B19] Ivanov D. A. Bernards M. A. (2016). Chlorophyll fluorescence imaging as a tool to monitor the progress of a root pathogen in a perennial plant. Planta 243, 263–279. doi: 10.1007/s00425-015-2427-9 26537710

[B20] Kalaji H. M. Schansker G. Brestic M. Bussotti F. Calatayud A. Ferroni L. . (2017). Frequently asked questions about chlorophyll fluorescence, the sequel. Photosynthesis Res. 132, 13–66. doi: 10.1007/s11120-016-0318-y PMC5357263 27815801

[B21] Kim J. H. Bhandari S. R. Chae S. Y. Cho M. C. Lee J. G. (2019). Application of maximum quantum yield, a parameter of chlorophyll fluorescence, for early determination of bacterial wilt in tomato seedlings. Horticulture Environment Biotechnol. 60, 821–829. doi: 10.1007/s13580-019-00182-0

[B22] Kingma D. P. Ba J. (2014). Adam: A method for stochastic optimization. arXiv preprint arXiv:1412.6980. doi: 10.48550/ARXIV.1412.6980

[B23] Kolber Z. S. Prášil O. Falkowski P. G. (1998). Measurements of variable chlorophyll fluorescence using fast repetition rate techniques: defining methodology and experimental protocols. Biochim. Biophys. Acta (BBA) - Bioenergetics 1367, 88–106. doi: 10.1016/S0005-2728(98)00135-2 9784616

[B24] Lefebvre M. Langrell S. Gomez y Paloma S. (2015). Incentives and policies for integrated pest management in europe: A review. Agron. Sustain. Dev. 35, 27–45. doi: 10.1007/s13593-014-0237-2

[B25] Lin K. Gong L. Huang Y. Liu C. Pan J. (2019). Deep learning-based segmentation and quantification of cucumber powdery mildew using convolutional neural network. Front. Plant Sci. 10. doi: 10.3389/fpls.2019.00155 PMC6413718 30891048

[B26] Liu J. Wang X. (2021). Plant diseases and pests detection based on deep learning: a review. Plant Methods 17, 1–18. doi: 10.1186/s13007-021-00722-9 33627131 PMC7903739

[B27] Li L. Zhang S. Wang B. (2021). Plant disease detection and classification by deep learning–a review. IEEE Access 9, 56683–56698. doi: 10.1109/ACCESS.2021.3069646

[B28] Lu Y. Young S. (2020). A survey of public datasets for computer vision tasks in precision agriculture. Comput. Electron. Agric. 178, 105760. doi: 10.1016/j.compag.2020.105760

[B29] Matous K. Benediktyová Z. Berger S. Roitsch T. G. Nedbal L. (2006). Case study of combinatorial imaging: What protocol and what chlorophyll fluorescence image to use when visualizing infection of *Arabidopsis thaliana* by *Pseudomonas syringae* ? Photosynthesis Res. 90, 243–253. doi: 10.1007/s11120-006-9120-6 17211582

[B30] Mavridou E. Vrochidou E. Papakostas G. A. Pachidis T. Kaburlasos V. G. (2019). Machine vision systems in precision agriculture for crop farming. J. Imaging 5, 89. doi: 10.3390/jimaging5120089 34460603 PMC8321169

[B31] Maxwell K. Johnson G. N. (2000). Chlorophyll fluorescence–a practical guide. J. Exp. Bot. 51, 659–668. doi: 10.1093/jexbot/51.345.659 10938857

[B32] Méline V. Brin C. Lebreton G. Ledroit L. Sochard D. Hunault G. . (2020). A computation method based on the combination of chlorophyll fluorescence parameters to improve the discrimination of visually similar phenotypes induced by bacterial virulence factors. Front. Plant Sci. 11. doi: 10.3389/fpls.2020.00213 PMC7055487 32174949

[B33] Méline V. Delage W. Brin C. Li-Marchetti C. Sochard D. Arlat M. . (2019). Role of the acquisition of a type 3 secretion system in the emergence of novel pathogenic strains of xanthomonas. Mol. Plant Pathol. 20, 33–50. doi: 10.1111/mpp.12737 30076773 PMC6430459

[B34] Minervini M. Fischbach A. Scharr H. Tsaftaris S. A. (2016). Finely-grained annotated datasets for image-based plant phenotyping. Pattern Recognition Letters 81, 80–89. doi: 10.1016/j.patrec.2015.10.013

[B35] Mohammad-Razdari A. Rousseau D. Bakhshipour A. Taylor S. Poveda J. Kiani H. (2022). Recent advances in e-monitoring of plant diseases. Biosensors Bioelectronics 201, 113953. doi: 10.1016/j.bios.2021.113953 34998118

[B36] Mutka A. M. Bart R. S. (2015). Image-based phenotyping of plant disease symptoms. Front. Plant Sci. 5. doi: 10.3389/fpls.2014.00734 PMC4283508 25601871

[B37] Pavicic M. Overmyer K. Rehman A. U. Jones P. Jacobson D. Himanen K. (2021). Image-based methods to score fungal pathogen symptom progression and severity in excised arabidopsis leaves. Plants 10, 158. doi: 10.3390/plants10010158 33467413 PMC7830641

[B38] Pérez-Bueno M. L. Pineda M. Barón M. (2019). Phenotyping plant responses to biotic stress by chlorophyll fluorescence imaging. Front. Plant Sci. 10, 1135. doi: 10.3389/fpls.2019.01135 31620158 PMC6759674

[B39] Rolfe S. Scholes J. (2010). Chlorophyll fluorescence imaging of plant-pathogen interactions. Protoplasma 247, 163–175. doi: 10.1007/s00709-010-0203-z 20814703

[B40] Ronneberger O. Fischer P. Brox T. (2015). U-Net: convolutional networks for biomedical image segmentation. In *Medical Image Computing and Computer-Assisted Intervention – MICCAI 2015* , eds. Navab N. Hornegger J.W. Wells M.A. Frangi F. (Cham: Springer International Publishing), 234–241.

[B41] Rousseau C. Belin E. Bove E. Rousseau D. Fabre F. Berruyer R. . (2013). High throughput quantitative phenotyping of plant resistance using chlorophyll fluorescence image analysis. Plant Methods 9, 17. doi: 10.1186/1746-4811-9-17 23758798 PMC3689632

[B42] Rousseau D. Chéné Y. Belin E. Semaan G. Trigui G. Boudehri K. . (2015b). Multiscale imaging of plants: current approaches and challenges. Plant Methods 11, 6. doi: 10.1186/s13007-015-0050-1 25694791 PMC4331374

[B43] Rousseau C. Hunault G. Gaillard S. Bourbeillon J. Montiel G. Simier P. . (2015a). Phenoplant: a web resource for the exploration of large chlorophyll fluorescence image datasets. Plant Methods 11, 24. doi: 10.1186/s13007-015-0068-4 25866549 PMC4392743

[B44] Sapoukhina N. Samiei S. Rasti P. Rousseau D. (2019). Data augmentation from RGB to chlorophyll fluorescence imaging application to leaf segmentation of arabidopsis thaliana from top view images. In 2019 IEEE/CVF Conference on Computer Vision and Pattern Recognition Workshops (CVPRW), (Long Beach, CA, USA: IEEE). 2563–2570. doi: 10.1109/CVPRW.2019.00312

[B45] Singh V. Sharma N. Singh S. (2020). A review of imaging techniques for plant disease detection. Artif. Intell. Agric. 4, 229–242. doi: 10.1016/j.aiia.2020.10.002

[B46] Sudre H. C. Li W. Vercauteren T. Ourselin S. Cardoso M. J. (2017). Generalised Dice overlap as a deep learning loss function for highly unbalanced segmentations. In Deep Learning in Medical Image Analysis and Multimodal Learning for Clinical Decision Support. (Springer International Publishing) 240–248. doi: 10.1007/978-3-319-67558-928 PMC7610921 34104926

[B47] Sun R. Zhang M. Yang K. Liu J. (2020). Data enhancement for plant disease classification using generated lesions. Appl. Sci. 10, 466. doi: 10.10.3390/app10020466

[B48] Valcke R. (2021). Can chlorophyll fluorescence imaging make the invisible visible? Photosynthetica 59, 381–398. doi: 10.32615/ps.2021.017

[B49] Wang H. Qian X. Zhang L. Xu S. Li H. Xia X. . (2018). A method of high throughput monitoring crop physiology using chlorophyll fluorescence and multispectral imaging. Front. Plant Sci. 9. doi: 10.3389/fpls.2018.00407 PMC5883069 29643864

[B50] Yakubovskiy P. (2019) Segmentation models. Available at: https://github.com/qubvel/segmentation_models.

[B51] Yuan H. Zhu J. Wang Q. Cheng M. Cai Z. (2022). An improved DeepLab v3+ deep learning network applied to the segmentation of grape leaf black rot spots. Front. Plant Sci. 13. doi: 10.3389/fpls.2022.795410 PMC8885525 35242151

[B52] Zhang H. Ge Y. Xie X. Atefi A. Wijewardane N. K. Thapa S. (2022). High throughput analysis of leaf chlorophyll content in sorghum using RGB, hyperspectral, and fluorescence imaging and sensor fusion. Plant Methods 18, 60. doi: 10.1186/s13007-022-00892-0 35505350 PMC9063379

[B53] Zhang K. Wu Q. Chen Y. (2021). Detecting soybean leaf disease from synthetic image using multi-feature fusion faster R-CNN. Comput. Electron. Agric. 183, 106064. doi: 10.1016/j.compag.2021.106064

